# Maternal N-Acetyl Cysteine Intake Improved Glucose Tolerance in Obese Mice Offspring

**DOI:** 10.3390/ijms21061981

**Published:** 2020-03-13

**Authors:** Michal Michlin, Lital Argaev-Frenkel, Liza Weinstein-Fudim, Asher Ornoy, Tovit Rosenzweig

**Affiliations:** 1 Departments of Molecular Biology and Nutrition Sciences, Ariel University, Ariel 40700, Israel; michal.michlin@gmail.com (M.M.); litalfren@gmail.com (L.A.-F.); 2 Laboratory of Teratology, Department of Medical Neurobiology, Hebrew University Hadassah Medical School, Jerusalem 91120, Israel; lizamami@walla.co.il (L.W.-F.); asher.ornoy@mail.huji.ac.il (A.O.); 3 Adelson School of Medicine, Ariel University, Ariel 40700, Israel

**Keywords:** glucose intolerance, high fat diet, metabolic programming, *N*-acetyl-l-cysteine, offspring, type 2 diabetes

## Abstract

Exposure to certain environmental factors during the early stages of development was found to affect health in adulthood. Among other environmental factors, oxidative stress has been suggested to be involved in fetal programming, leading to elevated risk for metabolic disorders, including type 2 diabetes; however, the possibility that antioxidant consumption during early life may affect the development of diabetes has scarcely been studied. The aim of this study was to investigate the effects of *N*-acetyl-l-cysteine (NAC) given during pregnancy and lactation on the susceptibility of offspring to develop glucose intolerance at adulthood. C57bl6/J mice were given NAC during pregnancy and lactation. High fat diet (HFD) was given to offspring at an age of 6 weeks for an additional 9 weeks, till the end of the study. Isolated islets of NAC-treated offspring (6 weeks old, before HFD feeding) had an increased efficacy of glucose-stimulated insulin secretion and a higher resistance to oxidative damage. Following HFD feeding, glucose tolerance and insulin sensitivity of NAC-treated offspring were improved. In addition, islet diameter was lower in male offspring of NAC-treated mice compared to their HFD-fed littermates. NAC consumption during early life improves glucose tolerance in adulthood in mice.

## 1. Introduction

The global prevalence of diabetes is >6%, with adult pre-diabetic estimates at 35% (USA data) [1]. Even more concerning, the number of affected individuals is still rising. Due to its severe complications, type 2 diabetes (T2D) is recognized as a pandemic, with major public health concerns. Therefore, prevention of T2D or delaying its onset is of high priority. 

T2D is a multifactorial disease developed in susceptible subjects as a result of exposure to various environmental cues. The most characterized diabetogenic factors are excess calorie intake, consuming a western diet and a sedentary lifestyle [2]. In addition, exposure to various additional environmental factors increases the susceptibility to develop glucose intolerance and T2D [3]. Oxidative stress is one of these diabetogenic factors, which has a central role in the development and progression of T2D and its associated comorbidities [4,5]. Antioxidant therapy, such as the administration of vitamin E or pentoxifylline, neutralized oxidative stress, improved glucose homeostasis and reduced the severity of nonalcoholic steatohepatitis (NASH) [6,7,8].

The impact of pathological environmental factors is higher when exposure occurs during critical periods, mainly early developmental stages of life. The hypothesis of non-genetic transgenerational s to elevated risks of metabolic syndrome, T2D, and other disorders [9,10,11]. Accordingly, antioxidants might negate the adverse effect of intrauterine oxidative stress, although the effect of antioxidant consumption during pregnancy and lactation on the development of diabetes in offspring at adulthood has scarcely been studied. The benefit of antioxidant given during prenatal period was demonstrated in a previous study, showing that polyphenol antioxidant, given to normal rats during pregnancy, improved the outcomes of postnatal over-nutrition [12]. On the other hand, a growing body of evidence suggests that a tightly controlled exposure to the oxidative milieu *in-utero* is highly important for a proper fetal development [9]. Accordingly, antioxidant supplementation during critical developmental periods might be taken with caution, and the efficacy and safety of such strategy should be validated.

The aim of this study was to evaluate whether maternal antioxidant supplementation during pregnancy and lactation might induce a positive metabolic programming of the offspring in view of the risk of developing diet-induced glucose intolerance and insulin resistance at adulthood. This was investigated using the antioxidant N-acetyl cysteine (NAC), given during pregnancy and lactation to healthy female mice, while the development of glucose intolerance was followed in high fat diet (HFD)-fed offspring.

## 2. Results

**Figure 1:**
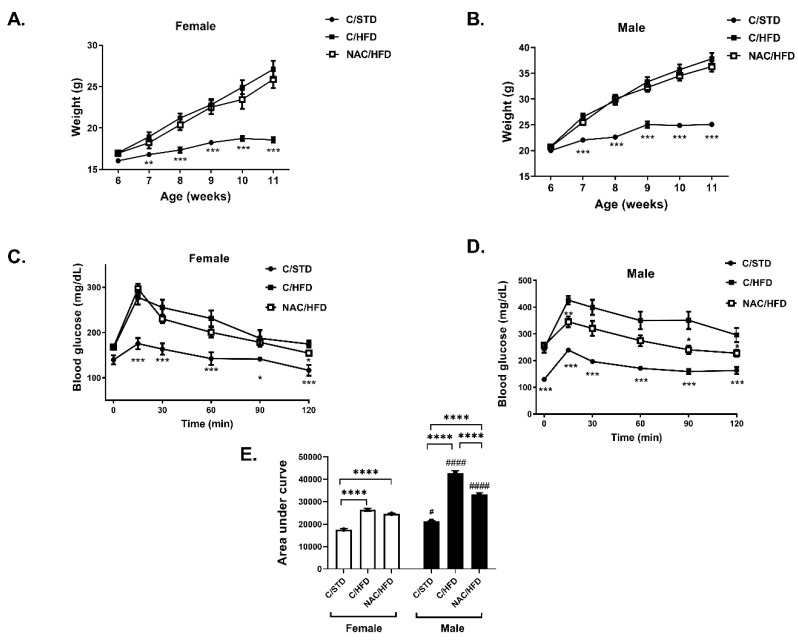
*N*-acetyl cysteine (NAC) improved glucose tolerance in high fat diet (HFD)-fed offspring of NAC-treated male mice. NAC was given to female mice during pregnancy and lactation. HFD was given to offspring from the age of 6 weeks. Body weight of female (**A**) and male (**B**) offspring was measured every week. Glucose tolerance test (GTT) was performed at age of 14 weeks in female (**C**) and male (**D**) offspring as described in Materials and Methods. The results are presented as mean ± SE, * *p* < 0.05. ** *p* < 0.005, *** *p* < 0.0005 compared to C/HFD mice, by one-way (**A**,**C**) or two-way (**C**,**D**) Anova. (**E**) Area under curve was calculated using GraphPad Prism 8. C/STD: STD-fed offspring of control mice, C/HFD: HFD-fed offspring of control mice, NAC/HFD: HFD-fed offspring of NAC-treated mice. The results are presented as mean ± SE (*n* = 8), **** *p* < 0.0001, # *p* < 0.05 and # *p* < 0.05, #### *p* < 0.0001 between genders, by two-way Anova followed by Tukey’s post-test.

NAC was given to female C57bl6/J mice (F_0_) during pregnancy and lactation and HFD was given to 6 week old offspring (F_1_). Body weight accumulation in F1 mice was measured: a higher body weight gain was found in HFD-fed compared to standard diet (STD)-fed mice, in both male and female, with males found to have higher body weight gain than females. NAC did not affect body weight of male and female offspring (Figure 1A,B). HFD feeding is known to induce a systemic insulin resistance leading to hyperinsulinemia and glucose intolerance. HFD feeding to the offspring increased fasting blood glucose and induced glucose intolerance in both female and male mice, as measured by glucose tolerance test (GTT) (Figure 1C–E), with males found to be much more susceptible to HFD-induced glucose intolerance than females. While fasting glucose was not affected by prenatal and early postnatal NAC treatment, glucose disposal following glucose load was significantly improved in male mice (Figure 1D,E). The effect of HFD feeding on glucose tolerance was lower in female compared to male mice, and was not affected by early exposure to NAC (Figure 1C,E). 

**Figure 2:**
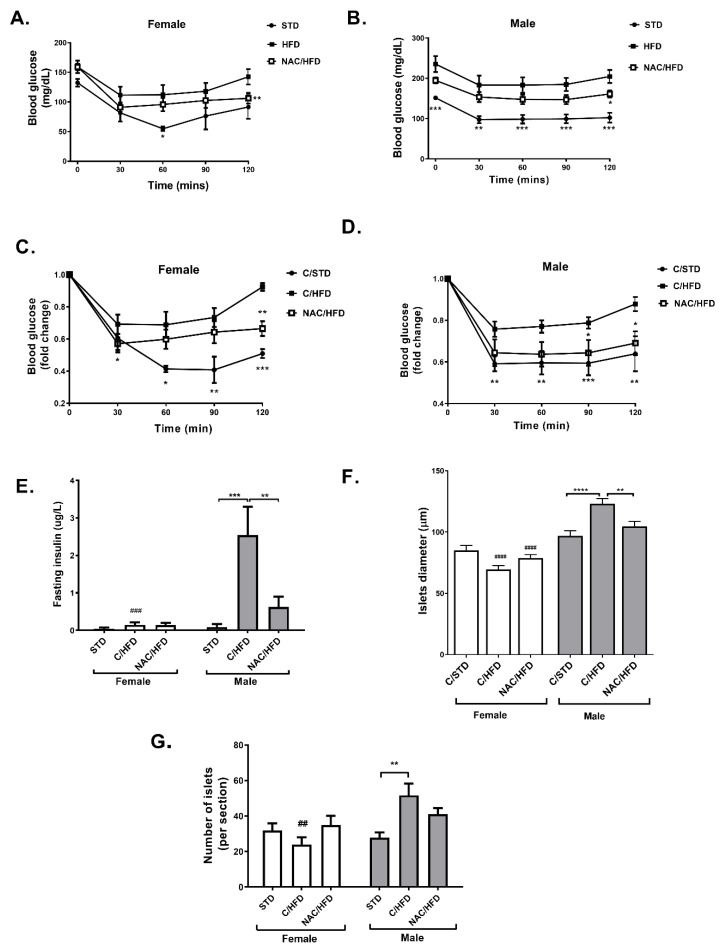
NAC improved insulin sensitivity in HFD-fed offspring of NAC-treated male mice. NAC was given to female mice during pregnancy and lactation. HFD was given to offspring from the age of 6 weeks. Insulin tolerance test (ITT) was performed at age of 15 weeks in female (**A**) and male (**B**) offspring as described in Methods. The results are presented as mean ± SE (*n* = 8), * *p* < 0.05, ** *p* < 0.005, *** *p* < 0.0005 by Student’s t-test, compared to C/HFD mice. Relative values are presented in (**C**) and (**D**). Fasting serum insulin was measured (*n* ≥ 8) at age of 16 weeks (**E**). H&E staining of pancreas was performed (5 mice, 3 section for each pancreas), and islet diameter (**F**) and number of islets per section (**G**) were measured. C/STD: STD-fed offspring of control mice, C/HFD: HFD-fed offspring of control mice, NAC/HFD: HFD-fed offspring of NAC-treated mice. The results are presented as mean ± SE, ** *p* < 0.005, *** *p* < 0.0005, **** *p* < 0.0005 and ## *p* < 0.005, ### *p* < 0.0005, between genders, in two-way Anova followed by Tukey’s post-test.

An insulin tolerance test demonstrated a disturbed insulin sensitivity in HFD-fed mice, with males found to be more insulin resistant than females (Figure 2A–D). Insulin resistance was improved in female NAC/HFD mice, and almost completely normalized in NAC/HFD males.

Fasting serum insulin was increased by HFD feeding in male mice, while early exposure to NAC abrogated this hyperinsulinemia (Figure 2E). Islet diameter and islet number were measured in sections of H&E stained pancreata of 17 weeks old offspring. In males, islet diameter was increased in C/HFD-fed mice, but not in NAC/HFD mice (Figure 2F). Similarly, the number of islets was higher in C/HFD, but not in NAC/HFD mice (Figure 2G). Surprisingly, an opposite effect was found in female mice: islet diameter was reduced by HFD feeding, an effect that was not found in offspring of NAC-treated mice. Similar results were found in the measurement of islet number. 

**Figure 3:**
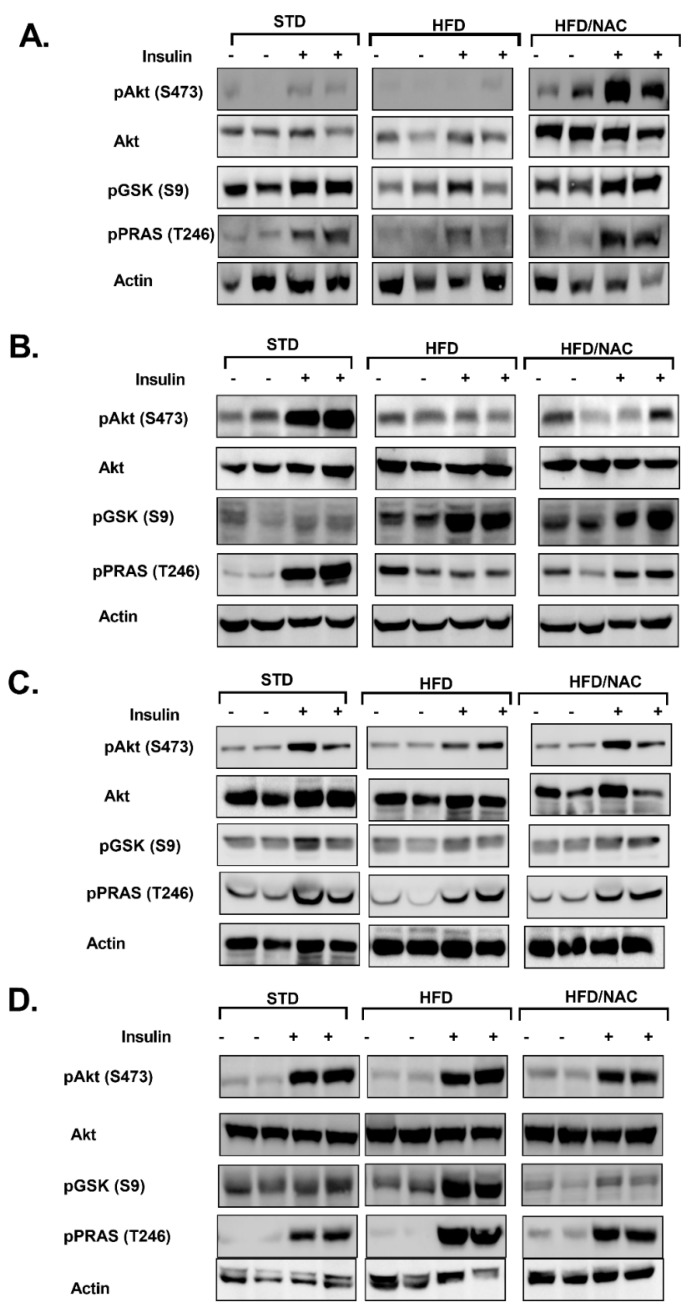
NAC improved the transmission of insulin signaling in HFD-fed offspring of NAC-treated male mice. NAC was given to females during pregnancy and lactation. HFD was given to offspring from the age of 6 weeks. Soleus muscle and liver of male (**A**,**B**, respectively) and soleus muscle and liver of female (**C**,**D**, respectively) offspring were removed at the age of 16 weeks as described in the Methods (*n* = 5). Western blot analysis was performed on protein lysates of muscle and liver using specific antibodies. C/STD: STD-fed offspring of control mice, C/HFD: HFD-fed offspring of control mice, NAC/HFD: HFD-fed offspring of NAC-treated mice.

Insulin sensitivity was investigated further by measuring the activation of insulin signaling cascade in the skeletal muscle and liver of offspring (Figure 3). Insulin resistance was developed in both the muscle and liver of HFD-fed male mice (Figure 3A,B); HFD feeding reduced insulin-induced phosphorylation of Akt, GSK3β and PRAS40, all of which are involved in the transmission of insulin pathways, while basal phosphorylation of GSK3β was enhanced by HFD feeding in the liver (Figure 3B). NAC improved insulin response in muscle, but not liver. Specifically, a marked increase in Akt phosphorylation was found in the muscle of NAC/HFD mice. Consistent with our previous observation of higher tolerance to HFD feeding in females (Figure 1 and Figure 2), insulin signaling was almost not affected in HFD-fed female mice (Figure 3C,D). Only a minor effect on insulin signaling, which was not improved by NAC, was found in muscle, as demonstrated by reduced insulin-induced Akt and PRAS40 phosphorylation (Figure 3C). Interestingly, insulin-induced GSK3β phosphorylation was significantly enhanced in the liver of HFD-fed female mice, an effect that was abrogated by NAC (Figure 3D). 

**Figure 4:**
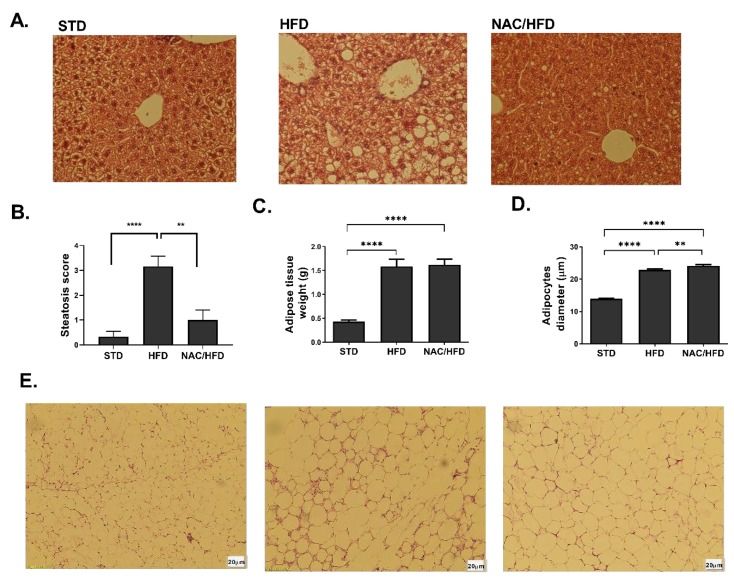
NAC reduced severity of hepatic steatosis and adipose tissue inflammation in HFD-fed offspring male mice. NAC was given to females during pregnancy and lactation. HFD was given to offspring from the age of 6 weeks. Mice were killed at age 16 weeks and H&E staining of liver (**A**) and adipose tissue (**E**) was performed. Severity of steatosis was graded using steatosis score, as described in Methods (**B**). Visceral adipose tissue weight was measured (**C**) and adipocyte diameter was measured (**D**). C/STD: STD-fed offspring of control mice, C/HFD: HFD-fed offspring of control mice, NAC/HFD: HFD-fed offspring of NAC-treated mice. The results are presented as mean ± SE (*n* ≥ 4), ** *p* < 0.005, **** *p* < 0.005 in one-way Anova followed by Tukey’s post-test.

Hepatic steatosis is one of the manifestations of insulin resistance and hyperinsulinemia. The severity of this condition was evaluated in sections of H&E-stained liver of male mice, using steatosis score (Figure 4A,B). A severe steatosis was found in C/HFD, covering over 66% of liver area. Almost complete normalization of liver histology was obtained in male offspring of NAC-treated mice. Adipocyte hypertrophy and adipose tissue inflammation is an additional characteristic of obesity and insulin resistance. Visceral adipose tissue mass was increased, and adipocyte diameter was enlarged in HFD-fed male mice, with no effect of early exposure to NAC on these parameters (Figure 4C,D). However, the number of crown-like structures in the tissue was significantly reduced in NAC/HFD mice (Figure 4E), demonstrating reduced infiltration of leukocytes toward the hypertrophic tissue. 

The results so far indicated that NAC supplementation during pregnancy and lactation reduced the susceptibility of offspring to the metabolic alterations induced by the HFD challenge. The improved insulin sensitivity of NAC-treated offspring minimized the effect of HFD feeding on islet mass and function.

**Figure 5:**
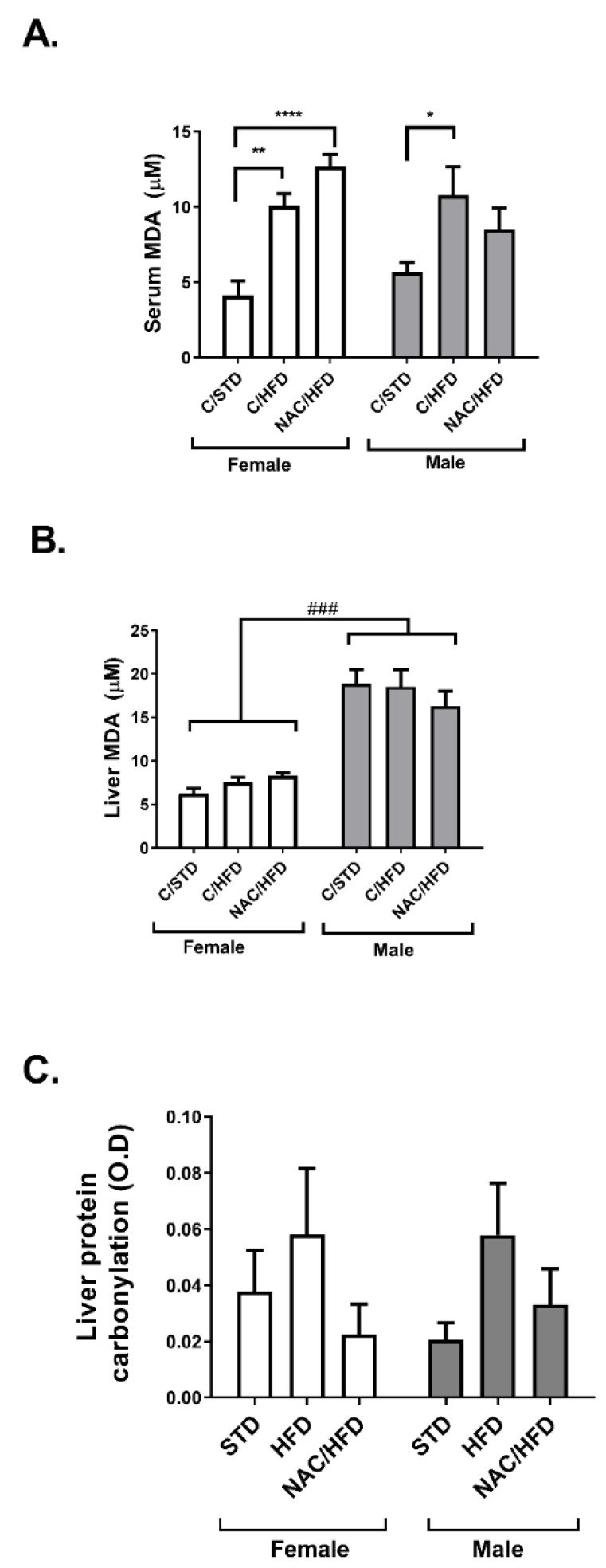
Effect of early life administration of NAC on markers of oxidative stress in HFD-fed mice. NAC was given to females during pregnancy and lactation. HFD was given to offspring from the age of 6 weeks. Mice were killed at age 16 weeks and serum (**A**) and liver (**B**) thiobarbituric acid reactive substrate (TBARS) were measured. (**C**) Protein carbonylation was measured in liver, as describes in Methods. The results are presented as mean ± SE (*n* = 8), * *p* < 0.05, ** *p* < 0.005, **** *p* < 0.0.0005 in one-way Anova followed by Tukey’s post-test. ### *p* < 0.0005 in two way Anova followed by Bonferroni’s post-test.

Recognizing its anti-oxidative properties, in the next set of experiments we measured the effect of NAC supplementation during pregnancy and lactation on the development of oxidative stress in the adult offspring, induced by HFD feeding. Lipid peroxidation was measured in serum and liver of STD and HFD-fed offspring of control and NAC-treated mice as a biomarker of oxidative stress (Figure 5A,B). The level of thiobarbituric acid reactive substrate (TBARS) was much higher in the liver of male compared to female offspring. While HFD feeding induced an elevation in the level of TBARS in the serum, but not in the livers of both males and females, NAC almost did not affect this biomarker of oxidative stress; only a minor effect was observed in serum level of TBARS. No significant difference was found between treatment groups in the level of protein carbonylation (Figure 5C).

**Figure 6:**
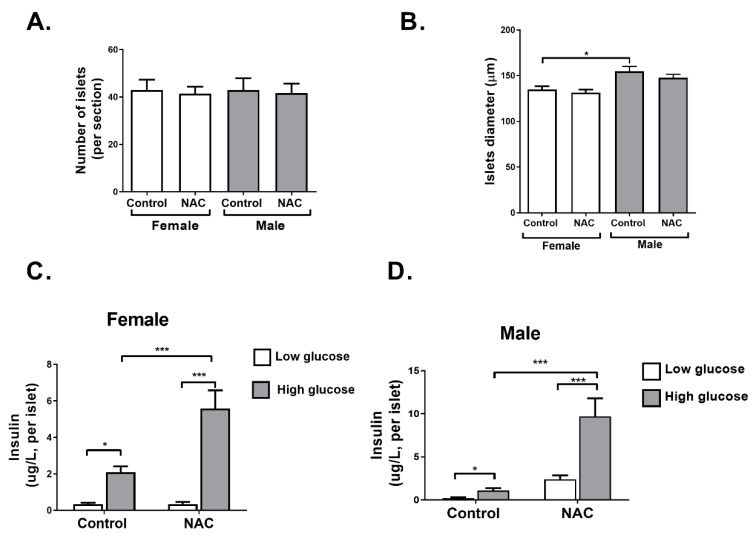
NAC improved pancreatic islet function in young offspring. NAC was given to females during pregnancy and lactation. Offspring were killed at age of 6 weeks, H&E staining of pancreas was performed, and number of islets per section (**A**) and islet diameter (**B**) were measured (5 mice, 3 sections for each pancreas). In additional set of 6 weeks old offspring, pancreatic islets were isolated and glucose stimulated insulin secretion (GSIS) was performed as described in Methods (**C**,**D**). The results are presented as mean ± SE (*n* = 6, 3 internal replicates for each experiment), * *p* < 0.05, *** *p* < 0.0005 in one-way Anova followed by Tukey’s post-test.

In the next part of the study, we aimed to investigate whether NAC supplementation during pregnancy and lactation might also exert a direct effect on pancreatic islet function in offspring, independent of HFD. To that effect, parameters of islet function were measured in offspring at age of 6 weeks, before the HFD regimen. Islet number and diameter were not changed in NAC offspring compared to controls in both male and female mice (Figure 6A,B). However, islet function, measured by glucose-induced insulin secretion assay, demonstrated a higher response of the islets to glucose stimulation, leading to increased insulin secretion in female and male offspring of NAC-treated mice (Figure 6C,D).

**Figure 7:**
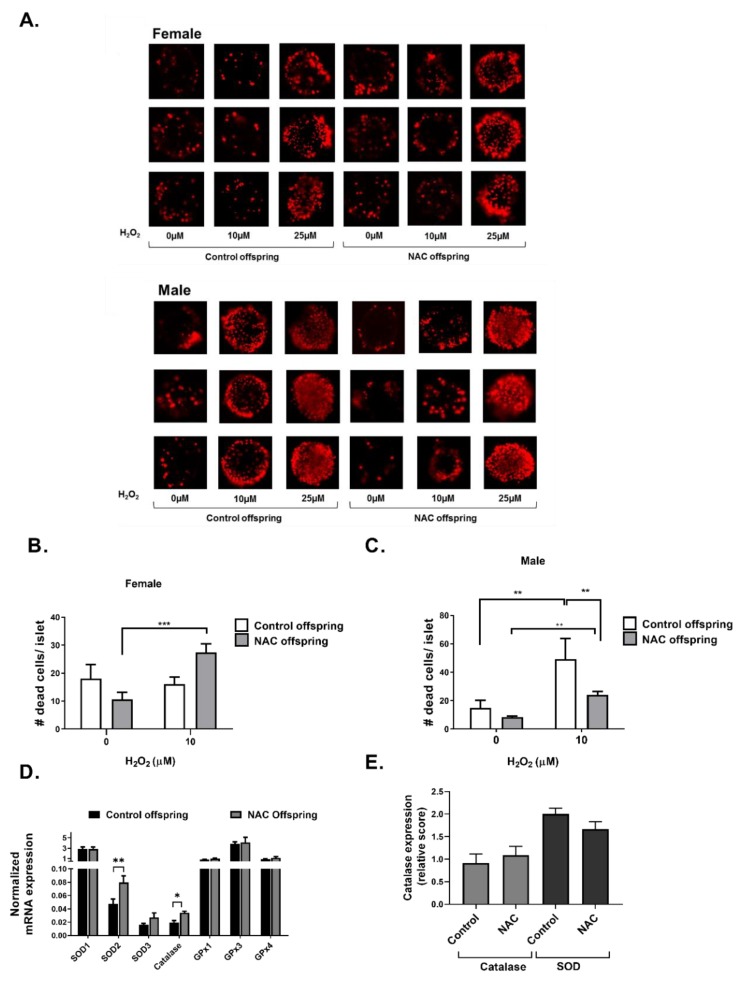
NAC increased the resistance of pancreatic islets against H_2_O_2_-induced oxidative stress. NAC was given to females during pregnancy and lactation. Pancreatic islets of offspring were isolated at age of 6 weeks. (**A**) Isolated pancreatic islets were treated with different doses of H_2_O_2_, and stained with propidium iodide, as described in Methods. A total of 3 representing micrographs are presented. The number of dead cells/islets was quantified in female (**B**) and male (**C**) mice. (**D**) mRNA was isolated from islets of 6 weeks old mice, and expression of selected genes was measured by RTPCR as described in Methods. Results were normalized to the expression of the housekeeping gene, RPS29 and are presented as the mean ± SEM. (**E**) Immunohistochemistry for the expression of SOD and Catalase was performed in pancreas of 6 weeks old male mice, and the expression level was blindly scored. The results are presented as mean ± SEM (*n* = 5, 3 replicates for each experiment), * *p* < 0.05, ** *p* < 0.005 and *** *p* < 0.0005 in one-way Anova followed by Tukey’s post-test.

As pancreatic islets are known to be highly sensitive to oxidative stress, we next measured whether NAC given during early development affects the capability of islets to resist oxidative stress. Isolated islets of 6 week old mice were exposed to increasing concentrations of H_2_O_2_, and cell viability was measured. Representing micrographs of PI-stained islets are presented in Figure 7A, and quantification of PI-stained cells/islets in female and male mice are presented in Figure 7B,C, respectively. Treatment with H_2_O_2_ concentrations higher than 25 µM induced an extensive cell death in the islets (data not shown). Islets of male mice were found to be more sensitive to oxidative stress than female, demonstrating an elevated rate of death in 10 µM H_2_O_2_, which was the lowest dose of H_2_O_2_ used in this experiment. However, higher resistance to H_2_O_2_ was found in offspring of NAC-treated male mice. NAC did not affect viability of islets isolated from female offspring. In order to find whether early exposure to NAC affects the expression of antioxidant enzymes in pancreatic islets, mRNA and protein expression of antioxidant enzymes were measured in male mice. As presented in Figure 7D, mRNA expression of Catalase and super oxide dismutase 2 (SOD2) in isolated pancreatic islets was increased following early exposure to NAC. However, these changes were not observed by immuno-histochemical staining of the pancreas (Figure 7E).

## 3. Discussion

This study demonstrated that early exposure to NAC programs offspring to more effectively accommodate metabolic stress induced by HFD at adulthood, leading to lower severity of insulin resistance. Our data also demonstrated that pancreatic islets are direct targets of the early treatment with NAC, as islet function was affected also in young mice, both male and female, before the administration of HFD feeding. Islet function, as evaluated by the capability to secrete insulin under conditions of high glucose, was improved in islets isolated from both male and female offspring of NAC-treated mice, suggesting a better adaptive response of β cells to conditions of hyperglycemia. In addition, the capability of pancreatic islets to resist oxidative stress was increased in male offspring of NAC-treated mice.

Our study demonstrated that the severity of metabolic alterations induced by HFD were gender specific, with female and male mice responding differently to HFD feeding. Insulin sensitivity and glucose tolerance were negatively affected by HFD feeding in males, while these metabolic alterations were significantly limited in HFD-fed females. This higher susceptibility to develop insulin resistance under conditions of energy oversupply were manifested by a set of alterations found in male mice: reduced glucose disposal rate following glucose load, lower response to insulin, hyperinsulinemia and islet hypertrophy. In females, HFD induced lower severity of glucose intolerance and insulin resistance, thus no hyperinsulinemia and islet hypertrophy were detected. These findings are consistent with previous studies performed on mice [13,14], demonstrating that sexual dimorphism exists in the response to HFD feeding in a large range of common metabolic variables. In addition, for the majority of variables, males were more affected than females by this diet. These results are also reflected by the significant difference in morbidity rate between human male and female in several pathologies associated with metabolic alterations [15,16]. 

NAC consumption during pregnancy and lactation improved glucose tolerance and insulin sensitivity in HFD-fed mice at adulthood. These beneficial outcomes of the intervention were more pronounced in male offspring, which were in general more negatively-affected by the diet. Insulin signaling in the muscle and liver of HFD-fed male mice was significantly disrupted compared to STD-fed male mice, demonstrated by reduced AKT and PRAS40 phosphorylation, while GSK3β phosphorylation was enhanced in livers of HFD-fed mice, in both basal and insulin-induced states, indicating the activation of pathways facilitating the storage of the surplus energy as glycogen. NAC consumption during pregnancy and lactation acquires some resistance against the diet-induced metabolic alteration, enabling better activation of insulin signaling in muscles of HFD-fed male mice. This beneficial effect of NAC was not found in livers of male offspring, suggesting a tissue specific effect of NAC. A tissue-specific response, with a distinct DNA methylation pattern, was observed in several models of prenatal exposure to stressors [17,18,19]. Uncovering the DNA methylome in muscle, liver and pancreatic islets might shed light on the tissue-specific outcomes of prenatal NAC administration. 

The insulin resistance induced by HFD feeding stimulates the secretion of higher levels of insulin in an effort to compensate for the lower response of target tissues to the hormone. As expected [20], islet hypertrophy developed due to this chronic hyperinsulinemia, as found in HFD-fed male mice, and normalized in offspring of NAC-treated mice which had improved insulin sensitivity. The expansion of β-cell might be attributed not only to islet hypertrophy, but also to neogenesis of islets, leading to an increase in their number, as found in HFD-fed male mice and as also reported previously [21]. Such an increase in islet number was not detected either in HFD-fed female mice or in male offspring of NAC-treated mice, which had a better sensitivity to insulin. Of high interest are the results demonstrating the sensitivity of isolated islets to oxidative stress. Islets of control male mice were more sensitive than female islets to H_2_O_2_. This is consistent with the findings in both rodents and humans, demonstrating a gender difference in oxidative stress, with females characterized by lower levels of oxidative stress than males [22]. Reactive oxygen species (ROS) production was lower and activity of antioxidant enzymes was higher in females compared to males [23,24]. Accordingly, NAC administration during pregnancy and lactation increased the resistance of male islets to oxidative stress, while females were not affected, suggesting that NAC is involved in the programming of the antioxidant defense mechanisms, which enables better accommodation with oxidative stress. NAC increased mRNA expression of SOD2 and catalase, although the protein level of these enzymes was not changed. Such discrepancy between mRNA and protein expression has often been found, suggesting a different rate of transcription, translation and degradation [25]. We suggest that the higher level of mRNA expression of antioxidant enzymes enables the improved response of islets once oxidative stress is developed. It is well-known [26] that pancreatic β cells are vulnerable to oxidative damage as a result of inherited low activity of antioxidant enzymes in these cells. Thus, metabolic programming that improves the efficiency of the antioxidant defense mechanisms of pancreatic islets might enable β cells to cope more effectively with oxidative stress, driven by chronic hyperglycemia and hyperlipidemia [4,27] and to increase their survival. In addition to its effect on the antioxidant machinery, it is possible that NAC programs the activity of other systems required to resist metabolic stress. Accordingly, the effects of NAC on the sensitivity of islets to other stressors, such as inducers of endoplasmic reticulum (ER) stress or pro-inflammatory cytokines, should be investigated in future studies. 

Our results indicated that early treatment with NAC not only prevented insulin resistance, but also improved the functionality and survival of beta cells. Although insulin resistance is considered as the precursor pathology of T2D, pancreatic beta cell dysfunction plays a significant role in the deterioration of the non-clinical metabolic alteration toward overt T2D. In contrast to non-diabetic obese individuals, having their beta-cells adequately compensating for the resistance, a reduction in beta cell function and mass was observed in obese-diabetic individuals [20,28]. In addition, most genetic risk variants for T2D act through impairing insulin secretion rather than insulin action [29,30], supporting the role of beta cell abnormalities as a critical contributing pathology in T2D. Accordingly, our results demonstrating that maternal consumption of NAC had beneficial effects on both components of glucose homeostasis (insulin action in target tissues and beta cell function) are promising in view of developing a new strategy to fight the emerging pandemic of T2D. Supporting our conclusion that certain factors might beneficially program the fetus toward improved metabolic function in adulthood, it was previously demonstrated, that administration of metformin to normoglycemic female mice during gestation, induced an elevation in pancreatic β cell mass and insulin secretion capabilities in offspring [31]. The outcome of prenatal metformin administration on the response to HFD-feeding was not investigated, and remains an issue that should be addressed. 

Increasing evidence suggests that certain acquired characteristics might be passed on to the next generation. This concept of trans-generational inheritance might be one of the reasons for the alarming increase in the prevalence of T2D over generations, suggesting a vicious cycle of disease transmission. Previous studies have shown that poor maternal diet, both over- and under-nutrition, and exposure to certain endocrine disruptors increase risk of offspring developing metabolic diseases at adulthood [32,33,34,35]. Characterizing specific deleterious *in-utero* environmental factors and developing strategies to eliminate their exposure might lead to breaking this negative cycle of inheritance. In addition, several dietary ingredients supplemented *in-utero* and during the lactation period (i.e., resveratrol, omega-3 fatty acids and inositols [36,37,38,39]) were found to partially prevent adverse outcomes of poor maternal diet in the adult offspring. One mechanism suggested to mediate the benefits of some of these supplements was by negating diet-induced oxidative stress. Oxidative stress plays a key role in the pathophysiology of several pregnancy-related disorders such as preeclampsia and intra-uterine growth restriction [40,41,42]. Adequate maternal antioxidant status might negate these adverse outcomes of oxidative stress, as was found in several studies using vitamin C and E, demonstrating improved fetal outcome in diabetic pregnancy in rats [43], although the results of other studies were not conclusive [44,45]. Similarly, NAC might be recommended as a supplement with beneficial outcomes on the metabolic status of the offspring. The antioxidant used in this study is well recognized as a safe agent, either when given at adulthood [46] or during pregnancy [47,48]. Thus, although additional studies are required in order to further support its efficacy and safety, the high safety profile of NAC supports the potential of implicating the results of this study in the clinics. 

The mechanisms mediating the effects of NAC on metabolic programming should be investigated further. Oxidative stress plays a minor role in the pathology of glucose intolerance in HFD-fed mice, and only a minor increase in biomarkers of oxidative stress were found in adult HFD-fed mice. However, NAC might regulate the redox balance in the placenta and in the developing fetus, as reported before [49,50], leading to long-lasting consequences. The Keap1/Nrf2/ARE pathway might mediate the observed effects of early NAC administration. Under an oxidized state, Nrf2 separates from the cytosolic Keap1, and is translocated to the nucleus where it binds to antioxidant response elements (ARE) on the DNA and promotes the transcription of numerous genes. Target genes of Nrf2 were classified either as cytoprotective (i.e., antioxidants and detoxifying genes), or as genes of the metabolic network [51]. Accordingly, while the cytoprotective aspects of Nrf2 function are beneficial, Nrf2 was also found to be involved in glycolytic switch, adipogenesis and metabolic reprogramming [52,53]. Acting as an antioxidant, NAC is supposed to inhibit the transcriptional activity of Nrf2. The effects of NAC on Nrf2 in the placenta and/or in the fetus and the transcriptional outcomes of such regulation should be investigated in future studies. Epigenetic mechanisms are also expected to be involved in the metabolic programming induced by NAC. The most probable epigenetic modification is methylation of cytosine nucleotides in DNA, which has a long-lasting effect on gene expression of affected genes. DNA methylation is carried out by the enzyme DNA methyl transferase (DNMT), and is regulated by the level of the methyl donor, S-adenosylmethionine (SAM), and the biochemical pathways that are involved in the regulation of its availability. SAM is synthesized from methionine, which is obtained from the diet or by the methylation of homocysteine by methyl synthase (MS) [54]. Depletion of cysteine diverts homocysteine to the trans-sulfuration pathway, leading to lower activity of MS, and eventually to a reduction in SAM levels [55]. Sulfur-poor diets force the synthesis of cysteine from methionine, alter DNA methylation and increase the predisposition to T2D [56]. Such a reduction in the cysteine pool might also result from oxidative stress that utilizes cysteine as a substrate for glutathione (GSH) synthesis [57]. Thus, an intimate link between redox regulation and DNA methylation exists, and emphasizes the importance of accurate regulation of redox balance and maintaining GSH levels during critical developmental periods. We suggest that NAC administration replenishes cysteine and glutathione pools, which enables sufficient levels of methionine as a substrate for SAM synthesis, supporting proper DNA methylation. This hypothesis should be investigated further in additional studies. 

In this study, maternal consumption of NAC was along both pregnancy and lactation periods. Additional study should be performed in order to further identify whether the prenatal or the early postnatal periods are the critical periods for NAC action. The underlying epigenetic mechanisms involved in the long-lasting effects of NAC were not investigated in this study, and should be clarified in the future.

## 4. Materials and Methods

### 4.1. Materials

NAC was purchased from Calbiochem. Insulin, proteases and phosphatases inhibitors, Collagenase-P and Histopaque were all purchased from Sigma (Rehovot, Israel). Anti-phospho Akt (S473), anti-Akt, anti phospho-GSK (S9), anti phospho IR (Y1150/1151), anti IR and anti phospho-PRAS40 (T246) were purchased from Cell-signaling Technology (Danver, MA, USA). Anti-β-tubulin was purchased from Abcam, and secondary antibodies (Peroxidase-AffiniPure Goat Anti-Mouse IgG antibody and Peroxidase-AffiniPure Goat Anti-Rabbit IgG antibody) were purchased from Jackson ImmunoResearch Laboratories (West Baltimore, PE, USA). 

### 4.2. Methods

This study was carried out in accordance with the recommendations in the Guide for the Care and Use of Laboratory Animals of the National Institutes of Health. The protocol was approved by the Committee on the Ethics of Animal Experiments of the University of Ariel (Permit Number: IL 76-09-15 and IL 137-06-17 permitted at September 2015 and June 2017, respectively). Animals had been anesthetized by ketamine+xylazine as required, and all efforts were made to minimize suffering. Animal House operates in compliance with the rules and guidelines of the Israel Council for Research in Animals, based on the US NIH Guide for the Care and Use of Laboratory Animals. 

C57Bl6/J mice were purchased from Envigo Laboratories (Israel). The mice were housed in an animal laboratory with a controlled environment of 20–24 °C, 45–65% humidity and a 12 h light/dark cycle. 

#### 4.2.1. Study Design

**Figure 8:**
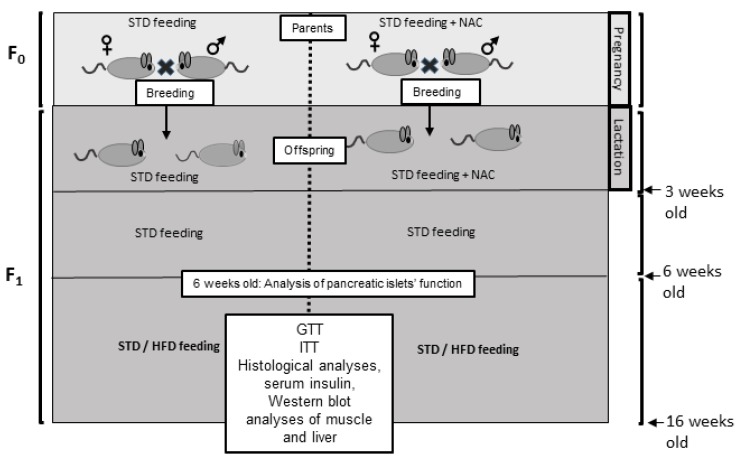
Study design. C57bl6/J mice were given STD with or without NAC supplementation during pregnancy and lactation. Offspring were separated at the age of 3 weeks. Analysis of islet mass and function was performed at age of 6 weeks. STD or HFD were given at age of 6 weeks till the end of the study.

The study was performed on a model of diet-induced glucose intolerance, using HFD-fed C57bl6/J mice. In this model, glucose intolerance is developed in C57bl6/J mice as a result of high fat diet feeding (HFD, 60% of total calories derived from fatty acids, 18.4% from proteins and 21.3% from carbohydrates. Harlan, Teklad TD.06414) at adulthood, starting at 6 weeks old till the end of experiments. Thus, in the models used in this study, offspring were developed in a healthy *in-utero* environment, and diabetes was developed during adulthood as a result of HFD feeding (see Figure 8 for a schematic description of the study design).

Breeding females (F0) were fed a standard diet, and were divided into 2 groups; control and NAC-treated, in which NAC at concentration of 400 mg/kg/day had been administered to females from breeding, through gestation and the lactation period. The dose used in this study was chosen according to our previous study, demonstrating beneficial effects of NAC on glycemic control in T2D mice, while no adverse effects were found [58]. Average consumption of water was measured and found to be 5 mL/day. NAC was dissolved in the drinking water so that the mice would receive NAC at about the dose of 400 mg/kg of their body weight daily. Neonates (F1) had been separated at 3 weeks of age, without additional supplementation of NAC. All offspring were fed STD during weeks 3–6. In one cohort, mice were killed at age of 6 weeks for pancreatic islet analyses, while in a second cohort, offspring were given either STD or HFD at age of 6 weeks till the end of the experiment at age 17 weeks, then mice were killed after a 6-h fast. In order to follow insulin-induced protein phosphorylation in liver and skeletal muscle, in some of the mice, insulin was injected (0.75 mU /g body weight) 15 min before killing the animal. Liver and muscle were snap frozen in liquid nitrogen, and preserved in −80 °C for later protein extraction. Part of the livers was saved in 4% paraformaldehyde for histological analyses. 

#### 4.2.2. Glucose and Insulin Tolerance Test

Intraperitoneal glucose tolerance test (GTT) was performed at 15 weeks. Mice were injected with 1.5 mg glucose/g body weight after a 6-h fast. Blood glucose was determined from tail blood using the ACCU-CHEK Go glucometer (Roche, Germany). Insulin tolerance test (ITT) was performed one week later following a 6-h fast. Glucose was measured following intraperitoneal insulin injection (0.5 U/kg). 

#### 4.2.3. Western Blot Analysis

Protein lysates were prepared using RIPA buffer supplemented with protease and phosphatase inhibitors. The samples were homogenized and centrifuged at 14,000 rpm for 20 min. The supernatant was collected and protein concentration was measured using the Bradford method. Proteins (20 μg per lane) were separated by SDS-polyacrylamide gel electrophoresis and electrophoretically transferred onto nitrocellulose membranes. The membranes were blocked in 5% dry milk, incubated with the appropriate antibody solutions (5% BSA in 0.01% TBST, dilution of 1:1000) and proteins were immunodetected using the enhanced chemiluminescence method.

#### 4.2.4. Histochemistry

Livers and visceral adipose tissue were isolated, fixed in 4% paraformaldehyde and embedded in paraffin. Consecutive 4 μm sections were cut and stained with hematoxylin and eosin (H&E). In order to measure hepatic steatosis, a steatosis score was attained through blinded evaluation by a pathologist. Scoring of liver sections was carried out according to Modified Brunt criteria of staging and grading of non-alcoholic fatty liver disease (NAFLD) [59].

#### 4.2.5. TBARS Assay

A thiobarbituric acid reactive substrate (TBARS) assay was used to measure lipid peroxidation in serum and liver samples. This method is based on chemical condensation reaction between the lipid peroxidation product malondialdehyde (MDA) and thiobarbituric acid (TBA). The reaction was performed as described [58] in high temperature and acidic conditions (95 °C, 20% acetic acid), forming a TBA-MDA adduct that is read at 530-540nm. 

#### 4.2.6. Protein Carbonylation Assay

Protein carbonyl content was measured using a colorimetric assay which provides an indicator of protein oxidation. This method is based on the chemical reaction between 2,4-dinitrophenylhydrazine (DNPH) and the protein carbonyls. This reaction forms a Schiff base, producing the corresponding hydrazone, which can be quantified spectrophotometrically at an absorbance between 360-385 nm. The assay was performed using a commercial kit (Cayman chemicals, 10005020) according to manufacturer’s instructions.

#### 4.2.7. Immunohistochemistry of Pancreas

Paraffin-embedded sections of the pancreas were used for the study. To evaluate the oxidative stress level we followed the expression of the antioxidants MnSOD (superoxide dismutase 2) and Catalase. Briefly, tissues were deparaffinized and rehydrated. For antigen retrieval, tissues were incubated in Tris-EDTA buffer (pH 9.0) in 80 °C for one hour, followed by 10 min of cooling. Endogenous peroxidases were blocked using 0.3% H_2_O_2_ in Methanol for 10 min. Off-target antigens were blocked using 5% bovine serum albumin. Tissue sections were stained with the following primary antibodies diluted 1:100: Rabbit polyclonal Anti-SOD2/MnSOD antibody (abcam AB-ab13533) and Rabbit polyclonal Anti-Catalase antibody (abcam AB16731) for overnight. Peroxidase-conjugated secondary antibodies (ImmPRESS™ Universal Antibody Polymer Detection KIT, VECTOR) were used. HRP activity was visualized using a 3,3′-diaminobenzidine tetrahydrochloride (DAB) substrate with hematoxylin as a counter-stain. Negative control sections were used in the absence of the primary antibodies (PBS). Tissue sections were then dehydrated and mounted and the intensity of the immunostaining was given an arbitrary score from 0–3. Sections were evaluated using an Olympus BH2 microscope. The examiners evaluated the sections without knowing to which group they belonged.

#### 4.2.8. Islet Isolation

Pancreatic islets were isolated by collagenase digestion of exocrine tissue, as described [60]. Mice were anesthetized by ketamine/xylazin mixture and killed by cervical dislocation. Collagenase-P (1.4 mg/mL) was injected through the bile duct to the exocrine pancreatic ducts to the inflation of the pancreas. The pancreas was isolated and incubated for 15 min in 3 mL of collagenase solution at 37 °C. Cold RPMI medium was added to the mixture and samples were centrifuged in 4 °C at 200× *g* for 1 min, then supernatant was discarded. This washing step was repeated twice again. Islets were separated by gradient using Histopaque solutions and 20 min centrifugation in 4 °C at 1250× *g*. Separated islets were picked under a dissecting microscope into warm RPMI culture media and incubated in an incubator (37 °C, 5% CO_2_). Islet diameter was measured and glucose stimulated insulin secretion was performed following 24 h of recovery. 

#### 4.2.9. Glucose Stimulated Insulin Secretion (GSIS)

The function of the isolated islets was measured by measuring GSIS. In this assay, isolated islets were incubated in a plate with a basal working solution of 3.3 mM glucose in Krebs Ringer Buffer (KRB) buffer at 37 °C for 30 min. Then, islets were transferred to a second plate with the same warm basal glucose solution for an additional 30 min. Islets were transferred to vials (10 islets in each vial) containing low and high glucose solutions (3.3 and 16.7 mM, respectively), for one hour incubation at 37 °C with moderate shaking. After gentle spindown of the islets, supernatant was collected and stored at −80 °C for insulin measurement.

#### 4.2.10. Quantitative Determination of insulin

Quantitative determination of insulin was performed by ELISA assay (Mercodia, Upsala, Sweden), according to the manufacturer’s instructions. The results were measured using a microplate reader (Tecan) at a wavelength of 450 nm.

#### 4.2.11. Real time PCR

Total RNA was extracted from isolated islets (6 weeks old mice) using TRI reagent (Molecular Research Center, Inc. Cincinnati, OH) according to manufacturer instructions. RNA (3 μg) was reverse transcribed by oligo-dT priming (Stratascript 5.0 multi-temperature reverse transcriptase, Stratagene) according to manufacturer instructions. Real-time PCR amplification reactions were performed using SYBR Fast Universal Ready-mix Kit (Kappa Biosystems, WI, USA), using the MxPro QPCR instrument (Stratagene). Primers for real time PCR reactions were synthesized by Sigma, Israel. Primer sequences are listed in Table 1.

#### 4.2.12. Islet Viability under Oxidative Conditions

In order to detect islet viability and sensitivity to oxidant, isolated islets were transferred into 6 wells plate (4 islets/well) and treated with different concentrations of H_2_O_2_ (0, 10 µM, 25 µM, 50 µM, 100 µM) for 20 h. Then, islets were stained with propidium iodide for 2 min. Images of isolated stained islets were taken by confocal microscope using a X10 magnification lens and edited using the image J program (ex/em 535/617 nm). 

#### 4.2.13. Statistical Analysis

Values are presented as mean± SEM. Statistical differences between the treatments and controls were tested by unpaired two-tailed Student’s *t*-test or one-way analysis of variance (ANOVA), followed by Bonferroni’s post-test or two-way ANOVA followed by Tukey’s post-test when appropriate. Analysis was performed using the GraphPad Prism 8.0 software. A difference of *p* ˂ 0.05 or less in the mean values was considered statistically significant.

## 5. Conclusions

While oxidative stress during pregnancy is clearly related to various pathological outcomes, the benefits of maternal antioxidant supplementation was barely investigated. In this study, we were able to confirm the hypothesis that the antioxidant NAC is a factor inducing a positive trans-generation inheritance in view of the susceptibility to develop insulin resistance and glucose intolerance at adulthood. This study is unique in that the supplement was given to healthy mothers, which were fed a standard diet, in contrast to previous studies that were performed on models of diabetic mothers or maternal poor-diet. Our results show for the first time that early life consumption of NAC decreased the deleterious metabolic effects caused by HFD feeding of the adult. Our data are in accord with previous study demonstrating the benefits of polyphenols antioxidants given to healthy rats, on the response of offspring to overfeeding, induced by reducing litter size [12]. In addition, flavonoids given to healthy mice during pregnancy, improved the antioxidant capacity of the adult offspring [61]. Our results, as well as the above mentioned publications [12,62] demonstrated that maternal antioxidant supplementation might be beneficial for offspring health, even when given to healthy mothers, which are not expected to experience oxidative stress. 

## Figures and Tables

**Table 1:** Primer sequences.

Gene	Forward	Reverse
*Catalase*	CTGGTTGTCATGCATGCACA	TGACAAAATGCTTCAGGGCC
*GPx1*	CCATCTGAGGGGATTTTCCT	TTGGTGATTACTGGCTGCAC
*GPx3*	ACCAATACCTTGAACTGAATGCAC	AATTAGGCACAAAGCCCCCA
*GPx4*	AGTACAGGGGTTTCGTGTGC	TATCGGGCATGCAGATCGAC
*SOD1*	CGGATGAAGAGAGGCATGTT	CACCTTTGCCCAAGTCATCT
*SOD2*	GCGGTCGTGTAAACCTCAAT	GATCTGCGCGTTAATGTGTG
*SOD3*	AGAGGCGGACAACACACAAT	ACGCCAGACTTGTGCATCTT
*RPS29*	TCGTTGGGCGTCTGAAGGCAA	CGGAAGCACTGGCGGCACAT

## Abbreviations

ARE	Antioxidant response elements
DNMT	DNA methyl transferase
ER stress	Endoplasmic reticulum stress
GPx	Glutathione peroxidase
GTT	Glucose tolerance test
HFD	High fat diet
ITT	Insulin tolerance test
Keap1	Kelch Like ECH Associated Protein 1
MS	methyl synthase
NAC	N-Acetyl Cysteine
NRF2	Nuclear factor erythroid 2-related factor 2
PI	Propidium iodide
ROS	Reactive oxygen species
RPS29	Ribosomal Protein S29
SAM	S-adenosyl methionine
SOD	Superoxide dismutase
STD	Standard diet
T2D	Type 2 diabetes
